# An Experiential Training Approach to Improving Early Childhood Nutrition Environments: Evaluation of the Feeding Brighter Futures Workshop

**DOI:** 10.3390/nu18142214

**Published:** 2026-07-08

**Authors:** Kimberly R. Haynie, Marilyn Bailey, W. Jinnings Burruss, Martha Ravola, Sankar Devarajan, Teki Hunt

**Affiliations:** 1 Department of Human Sciences, University of Arkansas at Pine Bluff, Pine Bluff, AR 71601, USA; 2 Department of Human Sciences, Alcorn State University, Lorman, MS 39096, USA

**Keywords:** early childhood nutrition, professional development, health promotion

## Abstract

Background/Objectives: Early childhood is a critical period for establishing lifelong dietary patterns, and early learning environments play a central role in shaping children’s eating behaviors. However, many childhood educators receive limited training in evidence-based nutrition practices. This study evaluated the impact of the Feeding Brighter Futures workshop, an experiential, case-based training intervention, on participants’ nutrition-related knowledge, perceptions, and attitudes. Methods: Using a pre–post survey design, undergraduate and graduate students, faculty, and staff (*n* = 33 pre; *n* = 19 post) from universities in Mississippi and southeastern Arkansas completed electronic surveys before and after the workshop. Survey questions aimed to assess participants’ knowledge of nutrition-focused regulations pertaining to early learning centers and comprehension of USDA MyPlate recommendations. Results: Results showed a statistically significant increase in agreement that teachers should only consume snacks available to children and further strengthening of already positive attitudes toward involving children in snack preparation. Attitudes toward using food as a reward did not significantly change. Conclusions: These findings suggest that brief experiential training may improve alignment with evidence-based nutrition practices, though further research with larger samples is warranted.

## 1. Introduction

Early childhood is widely recognized as one of the most influential and sensitive periods for establishing lifelong patterns of health, growth, and development. During this stage, rapid physiological, cognitive, and behavioral changes occur, creating a critical window in which environmental influences, particularly nutrition, can exert lasting effects on metabolic health. Research in developmental health has consistently shown that early dietary exposures do not merely shape short-term growth outcomes but instead help establish behavioral and biological trajectories that persist into adolescence and adulthood.

One of the most frequently cited indicators of this developmental sensitivity is the period of adiposity rebound, which typically occurs between five and seven years of age and has been strongly associated with the risk developing obesity later in life [1]. Importantly, subsequent investigations suggest that indicators of obesity and unhealthy weight trajectories may begin to emerge even earlier. Evidence suggests that risk factors for obesity emerge early, with predictors identifiable as early as two years of age [2] while Yucel, Kinik, and Aka [3] argue that meaningful obesity prevention efforts should begin in infancy, around six months of age. Together, these findings underscore that the roots of obesity are often established well before children reach school age, making early childhood a particularly critical period for prevention.

Against this developmental backdrop, the rising prevalence of early childhood obesity represents a serious and growing public health concern. Over the past several decades, rates of obesity among preschool-aged children have more than double [4]. Current estimates indicate that approximately 15 percent of children between the ages of two and five in the United States are classified as obese [5]. Even more alarming, data from the National Health and Nutrition Examination Survey (NHANES) show that the rate of severe obesity among children doubled between 2013 and 2023 [5]. These trends are particularly concerning given the strong evidence that obesity established in early childhood is likely to persist into adolescence and adulthood, increasing the risk for a wide range of chronic health conditions.

At the same time, early childhood represents not only a period of heightened risk, but also a powerful opportunity for prevention. This opportunity is especially significant given that approximately two-thirds of children under the age of five attend an organized early care or education setting [6]. For many young children, these environments account for a substantial proportion of their daily food intake, exposure to new foods, and social learning around eating behaviors. Adoption practices such as modeling of healthy eating and nutrition learning activities are positively associated with children’s willingness to try new foods when staff are not present [7]. As a result, early learning centers and childcare programs play a central role in shaping children’s food preferences, eating habits, and attitudes toward nutrition.

Within these settings, early childhood educators and caregivers serve not only as supervisors of meals and snacks, but also as role models and architects of the food environment. Their knowledge, attitudes, and practices related to nutrition can directly influence children’s willingness to try new foods, their perceptions of healthy eating, and their long-term dietary behaviors. Recognizing this influence, state licensing requirements and national guidelines increasingly emphasize the importance of developmentally appropriate nutrition practices, healthy food exposure, and positive mealtime routines in early learning environments.

Despite the critical importance of this role, many current and future educators receive limited formal training in nutrition or in evidence-based strategies for promoting healthy eating behaviors among young children. The National Center for Education Statistics indicates that only 37% of elementary school teachers self-reported receiving formal nutrition training as a component of their undergraduate and/or graduate training and an additional 15% received nutrition training exclusively through in-service learning experiences [8]. While there were assessments of formal nutrition education in early childhood learning center educators, the N-size was too small to extrapolate to a larger population [9]. This gap highlights the need for intentional, experiential learning opportunities that build foundational nutrition knowledge, increase awareness of regulatory standards, and strengthen confidence in implementing nutrition-focused activities in early childhood settings. The present study addresses this need by examining the impact of the Feeding Brighter Futures workshop, an experiential, case-based learning intervention designed to enhance participants’ knowledge, perceptions, and attitudes regarding early childhood nutrition. This study highlights the potential value of experiential learning as a supplemental pedagogical approach for enhancing undergraduate students’ understanding of evidence-based nutrition practices in early learning settings. Consistent with Kolb and Kolb’s assertion that experiential learning is a process through which students transform experience into knowledge, the findings suggest that engaging students in applied nutrition education activities may strengthen their ability to translate formal nutrition concepts into practical strategies that support healthy eating behaviors among young children [10]. By situating this work within the broader context of child development, public health, and early education policy, the study seeks to contribute to ongoing efforts to promote early, preventive approaches to improving lifelong health outcomes.

## 2. Materials and Methods

### 2.1. Participants and Setting

Participants in this study included undergraduate students, graduate students, faculty and staff from higher education institutions in Mississippi and southeastern Arkansas who attended the Feeding Brighter Futures workshop. Participants were recruited by announcements posted in email listservs. Additionally, informational flyers were disseminated to academic advisors of students majoring in nutrition, kinesiology, physical education, health and human development and family studies. Workshop participation and survey completion was exclusive to individuals enrolled in or employed at institutes of higher education in Arkansas and Mississippi. A total of 33 individuals completed the pre-survey, including 15 students and 18 faculty or staff members. Participation in the workshop and associated research activities was entirely voluntary. Students who chose to present the learning activity during the workshop had the opportunity to receive one either an Apple watch or Apple airpods. No incentives were offered for survey completion.

All workshop activities were conducted during a single, structured session that was approximately 6 h in duration. Data collection procedures were designed to ensure participant anonymity. No identifying information was collected, and survey responses were not linked to individual participants. The study procedures were implemented as part of an educational training event and were focused on program evaluation and instructional improvement.

### 2.2. Study Design

Evaluators obtained informed consent from participants before they commenced the pre-survey and the post-survey. All survey instruments were submitted to UAPB’s IRB board and were deemed as exempt from formal approval. A pre–post survey design was used to evaluate changes in participants’ nutrition knowledge, perceptions, and attitudes related to early childhood nutrition following participation in the workshop. This design allowed for the assessment of immediate changes associated with the intervention. Both quantitative and qualitative data were collected using electronic surveys administered immediately before and after the workshop.

The study employed a mixed-methods approach, combining Likert-scale survey items to measure changes in knowledge and attitudes with open-ended questions to capture participants’ perceptions and reflections regarding nutrition and early childhood feeding practices.

### 2.3. Pre-Survey Instrument

Prior to the commencement of any instructional activities, participants completed an electronic pre-survey. The pre-survey and post-survey questionnaires were developed specifically to address the information presented during the Feeding Brighter Futures workshop. Accordingly, the survey instruments were designed as pilots to establish baseline measures of participants’ nutrition knowledge, attitudes, and perceptions related to early childhood nutrition.

The instrument included:

- Likert-scale questions assessing familiarity with nutrition terminology, basic nutrition concepts, and beliefs about nutrition practices in early learning environments.
- Open-ended questions exploring participants’ perceptions of nutrition and their beliefs regarding its role in early childhood growth and development.

Likert-scale items used a 5-point response format ranging from strongly disagree (1) to strongly agree (5). The pre-survey required approximately 5–10 min to complete. 

### 2.4. Workshop Intervention

Following completion of the pre-survey and participant introductions, attendees participated in a structured, case-based experiential learning activity. Participants viewed a video case study describing a three-year-old child enrolled in a full-day early learning center who exhibited a strong aversion to fruits and vegetables. They also listen to presentations from faculty at UAPB and Alcorn discuss minimum licensing requirements and best practices for snack preparation for early learning centers and information sessions about nutrition and USDA MyPlate. The information session period was approximately two hours in duration.

Participants were tasked with collaboratively designing a co-curricular, nutrition-focused learning activity appropriate for an early childhood classroom that would encourage children to explore, taste, and become more accepting of fruits and vegetables.

To support activity development, participants were provided with supplemental instructional materials, including:

- Information on USDA MyPlate guidelines.
- An overview of macro- and micronutrients and their role in child development.
- USDA-recommended fruit and vegetable serving sizes for young children.
- An overview of Arkansas Minimum Licensing Requirements for Early Learning Centers.

Participants were explicitly instructed that their proposed activities must:

1. Be developmentally appropriate,
2. Comply with applicable licensing regulations, and
3. Promote exposure to and consumption of nutrient-dense, whole foods.

Participants worked in small groups of approximately four individuals to design their activities. At the conclusion of the activity period, which was approximately 2 h in duration, each group presented their proposed intervention to the full audience, allowing for discussion, feedback, and comparison of approaches. Each presentation was scored based on a rubric that evaluated (1) comprehension of early learner’s nutritional needs, (2) participant’s ability to address the problem statement, (3) curriculum development, (4) lesson design, (5) evaluating student feedback, (6) compliance with Arkansas Minimum Licensing Requirements for Early Learning Centers and (7) presentation and communication. The group that obtained the highest score at the end of the workshop received Apple watches or Apple airpods.

### 2.5. Post-Survey Instrument

Immediately following the conclusion of the workshop activities and group presentations, participants completed an electronic post-survey. The post-survey mirrored the structure and content of the pre-survey and included:

- The same Likert-scale items assessing nutrition knowledge, beliefs, and attitudes.
- The same or similar open-ended questions exploring perceptions of nutrition and its role in early childhood development.

This parallel structure allowed for direct comparison of pre- and post-workshop responses to assess changes associated with the intervention.

### 2.6. Data Analysis

All survey data were collected anonymously. Quantitative data from the Likert-scale items were aggregated and analyzed using descriptive statistics, including means and response distributions. Pre- and post-survey responses were compared to assess changes in nutrition knowledge and attitudes following participation in the workshop. Missing data or unanswered questions were omitted from the statistical analyses.

For selected items, Welch’s *t*-tests were used to compare pre- and post-survey mean responses to account for unequal sample sizes between the two survey administrations. Data were analyzed using GraphPad Prism Software Version 10.02. Significance was set at *p* < 0.05.

Qualitative responses to open-ended questions were reviewed and analyzed using a basic thematic review approach to identify common themes, shifts in perceptions, and changes in participants’ conceptualizations of early childhood nutrition and nutrition education.

## 3. Results

### 3.1. Participant Characteristics

A total of 33 participants completed the pre-survey and 19 participants completed the post-survey. Of the 33 pre-survey respondents, 12 (36.36%) were undergraduate students, 2 (6.06%) were graduate students, 17 (51.51%) were faculty and/or staff, 1 (3.03%) identified as an alumnus, and 1 (3.03%) declined to indicate their affiliation.

Participants represented a diverse range of academic disciplines and professional backgrounds. Three respondents (9.09%) identified education as their primary field, nine (27.27%) identified human development and family studies, five (15.15%) identified agriculture, four (12.12%) identified health, kinesiology, or physical education, and four (12.12%) identified dietetics, nutrition, or food science. Nine respondents reported “other” fields of study or professional specialization.

### 3.2. Inclusion of Nutrition Content and Dietary Practices

Participants were invited to report how frequently nutrition-related content was incorporated into their lectures, coursework, or educational materials. This item was included to assess participants’ prior exposure to nutrition-related concepts and educational resources before participation in the workshop. Twelve respondents indicated that nutrition information was included in most of their materials, 15 reported that it was included in some materials, and six indicated that nutrition content was rarely or never included.

Participants were also asked about their personal fruit and vegetable consumption. The majority of respondents (*n* = 23) reported consuming fresh fruits and vegetables daily, eight reported consuming a few times per week, and one respondent reported consuming fruits and vegetables less than once per week.

### 3.3. Perceptions of Optimal Timing for Nutrition Education

Participants were asked to identify the most effective age range for initiating nutrition education, choosing from the options of 3–5 years, 7–9 years, 10–13 years, or after age 13. In the pre-survey, 30 respondents (90.9%) selected ages 3–5 years as the most appropriate time to begin nutrition education. In the post-survey, 18 of 19 respondents (94.74%) selected this age range, representing a 3.83% increase from the pre-survey.

**Figure 1:**
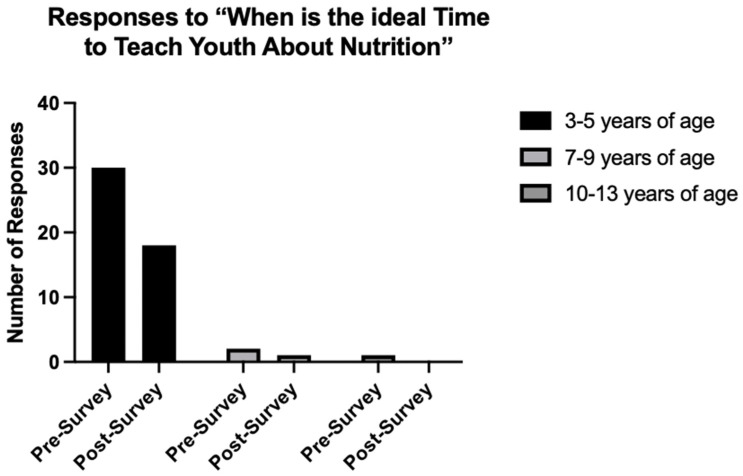
Workshop participants were asked to complete a pre-survey (*n* = 33) and post-survey (*n* = 19) questionnaire that included the question “When is the Ideal Time to Teach Youth About Nutrition?”. Respondents were asked to select one of the provided age ranges “3–5 years of age,” “7–9 years of age,” “10–13 years of age,” and “after 13 years of age.” The figure below illustrates the number of responses. A total of 30 of 33 pre-survey respondents (90.9%) vs. 18 of 19 post-survey respondents (94.74%) selected 3–5 years of age. Two of 33 pre-survey respondents (6%) vs. one of 19 post-survey respondents (5.26%) selected 7–9 years of age. One of 33 pre-survey respondents (3%) vs. no post-survey respondents (0%) selected 10–13 years of age. No respondents selected “after age 13” in either survey.

Two respondents (6.0%) in the pre-survey and one respondent (5.26%) in the post-survey selected ages 7–9 years. One respondent (3.0%) selected ages 10–13 years in the pre-survey, while no participants selected this option in the post-survey. No respondents selected “after age 13” in either survey. This data is illustrated in Figure 1.

### 3.4. Attitudes Toward Teacher Snack Consumption

Participants were asked to respond to the statement, “Teachers should only consume snacks that are also available to children in early learning centers,” a practice that is explicitly required under Arkansas Minimum Licensing Requirements and is intended to promote consistent behavioral modeling and equitable food environments within classrooms. Responses were recorded using a 5-point Likert scale ranging from strongly disagree (1) to strongly agree (5).

Analysis of the Likert-scale data revealed a meaningful and statistically significant shift in participants’ attitudes following the workshop. The mean pre-survey response was 3.7, indicating moderate agreement with the statement at baseline. In the post-survey, the mean response increased to 4.3, representing a mean increase of 0.62 (±0.26) points. A Welch’s *t*-test, used to account for unequal sample sizes between the pre- and post-survey groups, indicated that this increase was statistically significant (*p* < 0.05). This result suggests that participation in the workshop was associated with a stronger endorsement of the policy-aligned practice of teachers consuming only the same snacks that are available to children.

Examination of the response distributions further illustrates the nature of this shift. In the pre-survey, 12 respondents (36.4%) indicated that they “strongly agree” with the statement, while four respondents (12.1%) indicated that they “agree.” However, a substantial proportion of participants—12 respondents (36.4%)—selected “neither agree nor disagree,” and five respondents (15.2%) selected “disagree.” No participants selected “strongly disagree.” This distribution suggests that, prior to the workshop, a sizable portion of participants either held neutral views or were uncertain about the importance or appropriateness of this practice, despite its inclusion in state licensing regulations.

**Figure 2:**
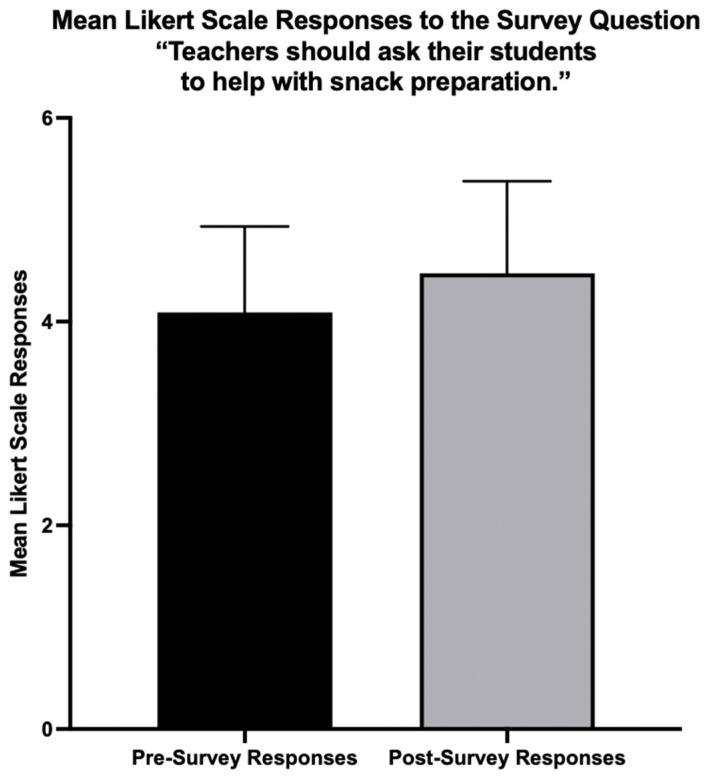
Workshop participants were asked to complete a pre-survey (*n* = 33) and post-survey (*n* = 19) questionnaire that included the question “Teachers should ask their students to help with snack preparation.” The mean pre-survey, coded, Likert-scale response was 4.09 (±0.83); the mean post-survey, coded, Likert-Scale response was 4.47 (±0.90). A Welch’s *t*-test was used to compare the mean of the coded survey responses (*p* = 0.70).

In contrast, post-survey responses suggest a shift toward stronger endorsement. Nine of the 19 respondents (47.37%) selected “strongly agree,” and seven respondents (36.84%) selected “agree.” Only three respondents (15.79%) selected “neither agree nor disagree,” and no respondents selected either “disagree” or “strongly disagree.” Compared to the pre-survey, this represents a marked reduction in neutral and negative responses and a consolidation of responses toward the agreement end of the scale. (Responses are outlined in Figure 2.)

These findings indicate that the workshop was associated not only with an increase in mean agreement scores, but also with a meaningful redistribution of responses away from uncertainty and disagreement and toward clear endorsement of this classroom practice.

### 3.5. Attitudes Toward Student Involvement in Snack Preparation

Participants were asked to respond to the statement, “Teachers should ask their students to help with snack preparation,” a practice that is encouraged and, in some cases, required under Arkansas Minimum Licensing Requirements as a means of promoting hands-on learning, food familiarity, and the development of healthy eating behaviors. Responses were recorded using a 5-point Likert scale ranging from strongly disagree (1) to strongly agree (5).

Analysis of Likert-scale responses indicated a positive shift in attitudes following participation in the workshop. The mean pre-survey response was 4.1, suggesting that participants already held generally favorable views toward involving children in snack preparation prior to the intervention. The mean post-survey response increased to 4.5, reflecting a mean increase of 0.38 (±0.25) points. Although this increase was not statistically significant based on a Welch’s *t*-test, the change indicates a strengthening of already positive attitudes.

Examination of response distributions provides additional insight into the nature of this shift. In the pre-survey, 13 of the 33 respondents (39.4%) indicated that they “strongly agree” with the statement, while 10 respondents (30.3%) indicated that they “agree.” However, a substantial proportion—10 respondents (30.3%)—selected “neither agree nor disagree,” suggesting some uncertainty or ambivalence about the practice despite overall favorable trends. No respondents selected “disagree” or “strongly disagree” in the pre-survey.

**Figure 3:**
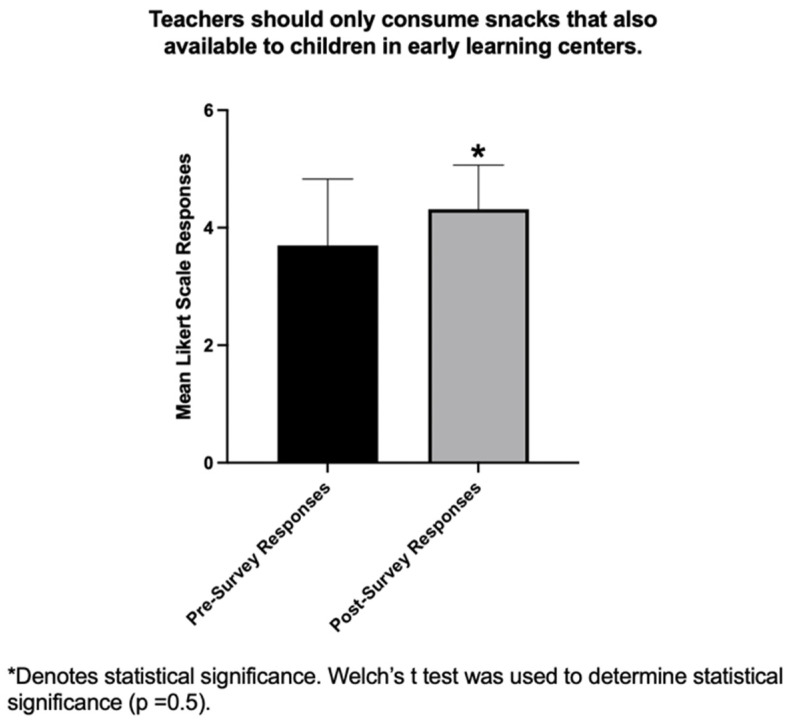
Workshop participants were asked to complete a pre-survey (*n* = 33) and post-survey (*n* = 19) questionnaire that included the question “Teachers should only consume snacks that are also available to children in early learning centers.” The mean pre-survey, coded, Likert-scale response was 3.69 (±1.11); the mean post-survey, coded, Likert-scale response was 4.32 (±0.75). A Welch’s *t*-test was used to compare the mean of the coded survey responses (*p* = 0.5).

In the post-survey, support for student involvement in snack preparation became more concentrated at the upper end of the scale. Thirteen of the 19 respondents (68.24%) selected “strongly agree,” and three respondents (15.79%) selected “agree.” Only two respondents (10.53%) selected “neither agree nor disagree,” and one respondent (5.26%) selected “disagree.” No respondents selected “strongly disagree.” Overall, these changes represent a 35.73% increase in the proportion of respondents who selected either “agree” or “strongly agree” compared to the pre-survey. (Responses are outlined in Figure 3.)

### 3.6. Attitudes Towards the Use of Food as a Reward

Participants were asked to respond to the statement, “Pizza parties are great ways to promote positive behaviors in classrooms,” a practice that is discouraged under Arkansas Minimum Licensing Requirements, which specify that food should not be used as a behavioral reward or incentive. Responses were recorded using a 5-point Likert scale ranging from strongly disagree (1) to strongly agree (5).

Analysis of Likert-scale responses revealed that participation in the workshop did not result in a change in opinions pertaining to this inquiry. The mean pre-survey response was 3.44 (±1.13), and the mean post survey response was 3.37 (±1.54). A Welch’s *t*-test was used to compare the mean of the coded survey responses (*p* = 0.87).

Closer examination of the response distributions provides additional insight into the nature of this shift. In the pre-survey, eight of the 33 respondents (25%) indicated that they “strongly agree” with the statement, and eight respondents (25%) indicated that they “agree.” Ten respondents (31.3%) selected “neither agree nor disagree,” two respondents (6.3%) selected “disagree,” and four respondents (12.5%) selected “strongly disagree.” This distribution indicates that, prior to the workshop, opinions were mixed, with a substantial proportion of participants expressing either support for or neutrality toward the use of food-based rewards.

**Figure 4:**
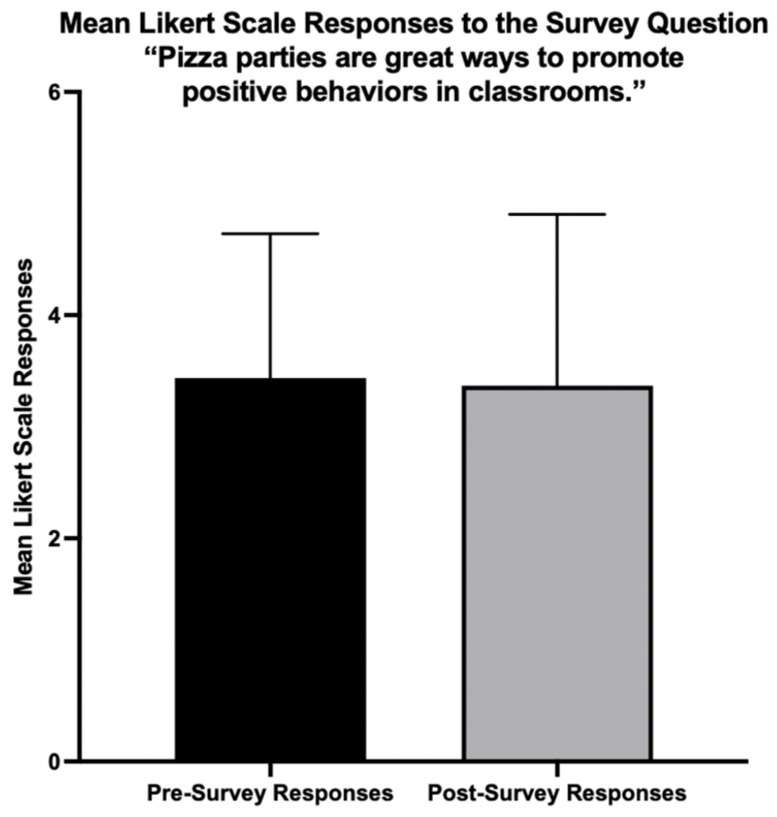
Workshop participants were asked to complete a pre-survey (*n* = 33) and post-survey (*n* = 19) questionnaire that included the question “Pizza parties are great ways to promote positive behaviors in classrooms.” The mean pre-survey, coded, Likert-scale response was 3.44 (±1.13); the mean post-survey, coded, Likert-Scale response was 3.37 (±1.54). A Welch’s *t*-test was used to compare the mean of the coded survey responses (*p* = 0.87).

In the post-survey, responses became more polarized. Seven of the 19 respondents (36.84%) selected “strongly agree,” while two respondents (10.53%) selected “agree.” Four respondents (21.05%) selected “neither agree nor disagree,” three respondents (15.79%) selected “disagree,” and three respondents (15.79%) selected “strongly disagree.” We did not observe quantitative differences in mean survey responses to attitudes and perceptions regarding use of food (pizza) as a reward. Additionally, we did not observe significant differences in the distribution of responses to the survey question (*p* = 0.70). (Responses are outlined in Figure 4.)

## 4. Discussion

The purpose of this study was to examine whether participation in the Feeding Brighter Futures experiential workshop was associated with changes in participants’ knowledge, perceptions, and attitudes related to early childhood nutrition and classroom feeding practices. Given the well-established importance of early childhood as a critical period for shaping lifelong dietary patterns and health trajectories, and the central role of early learning environments in children’s daily food experiences, strengthening the nutrition-related preparation of educators and related professionals represents an important upstream strategy for obesity prevention and health promotion. Participants represented an array of disciplinary fields, including agriculture, nutrition, human development and family studies and kinesiology. This disciplinary diversity suggests that the workshop reached participants with varying levels of prior exposure to nutrition-related content.

Overall, the findings suggest that the workshop was associated with meaningful shifts in several key attitudinal domains that are directly tied to early childhood nutrition policy and best practices. Participants displayed a statistically significant increase in agreement with the principle that teachers should only consume snacks that are also available to children. This shift is noteworthy because teacher modeling is a powerful and well-documented mechanism through which children acquire food preferences, eating behaviors, and attitudes toward healthy foods. However, there is inconsistent integration of best practices associated with nutrition in early learning settings. While previous studies have assessed formal nutrition education in pre-k educators, there is a dearth of data that evaluates co-curricular learning activities on nutrition knowledge and application in classroom settings. By strengthening participants’ endorsement of this practice, the workshop appears to have increased alignment between participants’ beliefs and some licensing standards, as well as with broader evidence-based recommendations for creating supportive nutrition environments in early learning settings.

Although not all attitudes shifted uniformly—most notably with respect to the use of food as a reward—the overall pattern of findings suggests that targeted, policy-aligned professional development can strengthen conceptual alignment between regulatory standards, evidence-based practice, and educators’ everyday decision-making. The question was framed to suggest that “pizza parties” equated to using energy-dense foods as a reward. However, in individual discussions, students verbalized that they may adjust the concept of “pizza party” to include nutrient-dense toppings and whole grain crusts. Accordingly, this question did not capture possible qualitative changes in students’ perceptions in the usage of food as a reward in early learning settings. In this sense, the results not only support the promise of the intervention, but also help identify priority areas for future training and policy emphasis.

Similarly, although participants already held generally favorable views toward involving children in snack preparation prior to the workshop, results indicate a further strengthening of these attitudes following the intervention. The substantial increase in the proportion of respondents who “strongly agreed” with this practice suggests increased confidence in the developmental and educational value of hands-on food involvement. This is particularly important because participation in food preparation has been shown to increase children’s willingness to try new foods, improve food familiarity, and support the development of self-regulation and autonomy around eating.

In contrast, attitudes toward the use of food as a behavioral model did not change following participation in the survey. There was a negligible decrease in mean survey responses. This finding suggests that beliefs about food-based rewards may be more deeply entrenched, culturally normative, or context-dependent than other classroom nutrition practices. It also highlights that some nutrition-related practices—particularly those tied to classroom management traditions and reward systems—may require more explicit, direct, and sustained professional development to meaningfully change.

Taken together, these findings suggest that training may improve alignment with evidence-based nutrition practices. Importantly, this intervention targeted not only current educators but also future professionals across a range of disciplines, suggesting that such training may have broad relevance across the early childhood workforce pipeline.

From a policy and systems perspective, these results support the inclusion of cocurricular, experiential learning experiences into teacher preparation programs, professional development offerings, and interdisciplinary training initiatives. Because early learning environments serve a majority of young children and account for a substantial portion of their daily food exposure, even modest improvements in educator practices and beliefs may have the potential to yield meaningful population-level impacts over time. However, more studies are needed to substantiate this claim.

Several limitations should be considered when interpreting the findings of this study. First, the sample size was relatively small. Additionally, there was a 43% attrition in respondents between the pre- and post-survey (33 vs. 19, respectively). The challenges in N size, and high attrition rates limit statistical power and reduce the ability to detect smaller but potentially meaningful effects. While we did observe statistically significant changes in one of the survey responses, the limited N-size of the study increased the possibility of both a Type I and Type II statistical error. Thus, the data points were less generalizable over the broader target population. The high attrition rate may be associated with the length of the workshop, which was approximately 6 h in duration. Most of the participants who left the workshop prior to the official conclusion time did so because of schedule conflicts (i.e., conflicting class schedules, etc.). Accordingly, student class schedules should be taken into consideration when planning future experiential learning activities. It also limits the generalizability of the findings beyond the specific institutions and regional context represented in the workshop.

Second, the study relied on self-reported attitudes and perceptions rather than direct observation of classroom practices or objective measures of behavior change. While attitudes are an important precursor to practice, future research should examine whether these shifts translate into sustained changes in educator behavior and classroom food environments.

Third, the study employed a short-term pre–post design that assessed immediate changes following the workshop. This design does not allow for conclusions about the durability of the observed attitude changes. Additionally, the study design does not assess the relationship between attitude changes and classroom implementation. Longitudinal follow-up studies are needed to determine whether these shifts persist over time and influence professional practice.

Fourth, the participant group included a mix of students, faculty, and staff from diverse disciplinary backgrounds. While this interdisciplinary composition is a strength in terms of reach, it also introduces variability in prior knowledge, professional roles, and relevance to daily practice that could not be fully controlled in the analysis. Early childhood education pedagogy is specifically focused on cognitive and physical development in young children. By engaging in multi-disciplinary groups, participants (specifically those majoring in human development) were challenged to expand their knowledge base and to ideate in ways that reflected interdisciplinary implementation of early childhood pedagogy.

Lastly, the post-survey concluded with an open-ended question about their takeaways from the activity. Six post-survey respondents indicated that the workshop reinforced that eating healthy foods is a lifelong practice. An additional respondent indicated that the workshop highlighted that “getting kids to try vegetables can be fun, and not a punishment.”

Future research should aim to replicate this intervention with larger and more diverse samples, including in-service early childhood educators across multiple states or systems. Studies should also incorporate behavioral and environmental outcome measures, such as classroom observations, menu analyses, or child food exposure metrics. Additionally, future interventions may benefit from placing greater emphasis on challenging entrenched practices such as the use of food as a reward, potentially through more explicit discussion of behavioral theory, classroom management alternatives, and ethical considerations related to food-based incentives.

## 5. Conclusions

The workshop led to improved alignment with evidence-based nutrition practices among early childhood educators, especially in supporting teacher modeling of health eating and involving children in snack preparation. However, attitudes toward using food as a reward did not change. Key limitations include a small sample size, high attrition, reliance on self-reported data, short-term assessment, and limited generaliza-bility due to participant diversity and regional context. 

## Data Availability

The raw data supporting the conclusions of this article will be made available by the authors on request.
